# The Russian Registry of Chronic Hypoparathyroidism

**DOI:** 10.3389/fendo.2022.800119

**Published:** 2022-02-16

**Authors:** Elena V. Kovaleva, Anna K. Eremkina, Alina R. Elfimova, Julia A. Krupinova, Ekaterina E. Bibik, Irina S. Maganeva, Anna M. Gorbacheva, Ekaterina A. Dobreva, Galina A. Melnichenko, Natalia G. Mokrysheva

**Affiliations:** Department of Parathyroid Glands Pathology, Endocrinology Research Centre, Moscow, Russia

**Keywords:** hypoparathyroidism, complications, prognosis, bone mineral density, database

## Abstract

**Introduction:**

Chronic hypoparathyroidism is a relatively rare disease associated with multicomponent medical therapy and various complications. The analysis of large databases of patients with chronic hypoparathyroidism is a necessary tool to enhance quality of medical care, as well as to determine the optimal clinical and therapeutic approaches, and prognostic markers of the disease.

**The Aim:**

of this study is to estimate the clinical and biochemical profile, long-term complications, medical therapy and disease control of the patients with chronic postsurgical and non-surgical hypoparathyroidism.

**Materials and Methods:**

the cross-sectional, observational, continuous study was based on the Russian Registry of patients with hypoparathyroidism. 544 patients from 63 regions of the Russian Federation were included in this study.

**Results:**

The majority of cases had postsurgical etiology (88.4%). Postsurgical hypoparathyroidism prevailed in females (р<0.001). About a half of patients had blood calcium and phosphorus targets, 56 and 52% respectively. Nephrolithiasis was confirmed in 32.5%, nephrocalcinosis - in 12.3% of cases. The risk of nephrocalcinosis/nephrolithiasis increased by 1.85 times with disease duration more than 4.5 years. The cataract was found in 9.4%. The cut-off point for the development of cataracts was 9.5 years, with a 6.96-fold increased risk. The longer duration of hypoparathyroidism of any etiology was associated with more frequent cataract (p=0.0018).We found brain calcification in 4%, arrhythmias in 7.2% and neuropsychiatric symptoms in 5.15% of cases. Generally, the BMD in the studied group corresponded to age values, and there was no evidence for the phenomenon of high bone density. TBS was consistent with normal bone microarchitectonics. In our study, the majority of patients (83.5%) was treated with standard therapy of calcium and vitamin D supplements. 5 patients with severe disease course were treated with rhPTH (1–34).

**Conclusions:**

Analysis of the presented database indicates insufficient diagnosis of the complications associated with chronic hypoparathyroidism. Overall, hypoparathyroidism is associated with higher risks of renal stone formation, decreased GFR, cataract especially in patients with longer duration of disease.

## Introduction

Hypoparathyroidism is a rare endocrine disease characterized by hypocalcemia due to absent or inappropriately low serum parathyroid hormone (PTH) levels. Clinical symptoms are various and broadly related to the severity of hypocalcemia. In case of a sharp decrease in blood calcium level, they can be acute, such as neuromuscular irritability, weakness, spasms/twitches or cramps. Or they can be chronic and affect a number of organs (1).

Hypoparathyroidism may be transient (postsurgical, functional) or chronic. Chronic hypoparathyroidism is more often caused by neck surgery (~75% of cases) resulting in injury to the parathyroid glands (PTG) or their blood supply and is closely related to the number of PTG remaining after surgery: 16% for cases with one to two preserved glands, 6% for three and 2.5% for four glands (2, 3). Postsurgical hypoparathyroidism is generally defined as chronic (permanent) when PTH secretion is insufficient to maintain normocalcemia 6-12 months after surgery. Autoimmune and rare genetic conditions may also cause hypoparathyroidism in approximately 20-25% cases, but some of them remain idiopathic (4). The epidemiological studies are insufficient. In the United States the estimated prevalence of chronic hypoparathyroidism is nearly 37 cases per 100,000 people, among them 8 cases per 100,000 have non-surgical etiology. In Denmark, the overall prevalence is similar to the results in the USA, around 24 per 100,000 people, with rates of 2.3 per 100,000 non-surgical hypoparathyroidism. The annual incidence of cases is around 0.8 per 100,000 people (5, 6). However, in Norway the prevalence of hypoparathyroidism based on hospital registry was lower at 10.2 per 100,000. In addition, there was a slight increase in the incidence of non-surgical hypoparathyroidism 3.0 per 100,000 versus postsurgical cases 6.4 per 100,000 (7). The primary analysis of the Endocrinology Research Centre (Moscow, Russia) database for patients with hypoparathyroidism was carried out in 2020 (8). The aim of this study was to conduct the initial analysis of clinical presentations and hospital management of patients with chronic hypoparathyroidism and for this, the Italian registry model was taken. Two hundred patients with hypoparathyroidism (n = 194) and pseudohypoparathyroidism (n = 6) were enrolled. There was a single-center study, moreover the pediatric patients were also excluded because the study was conducted in adult departments (8). Thus, this data could not represent the epidemiology of chronic hypoparathyroidism in the Russian Federation. This served as the basis for a fundamentally new approach – the creation of Russian online registry of chronic postsurgical and non-surgical hypoparathyroidism with the possibility of an access code in various regions of the country and, consequently, the recruitment of new patients.

Chronic hypoparathyroidism of any etiology requires lifelong multicomponent therapy, as well as careful monitoring and an individual approach to choose the optimal treatment strategy. In the absence of adequate follow-up, the risks of long-term complications significantly increase particularly in the renal, neuropsychiatric, skeletal, cardiovascular and musculoskeletal systems. Short-term and long-term complications of the disease often result in impaired health-related quality of life (QoL) (4). Evaluation of the epidemiological and clinical features of chronic postsurgical and non-surgical hypoparathyroidism is necessary to predict severe complications and to improve the quality of medical care.

The aim of this study is to estimate the clinical and biochemical profile, long-term complications, medical therapy and disease control of the patients with chronic postsurgical and non-surgical hypoparathyroidism.

## Materials and Methods

### Study design

The cross-sectional, observational, continuous study was conducted at the Endocrinology Research Centre (Moscow) and approved by the Ethics Committee (Protocol No. 18 dated October 11, 2017). The technical development and start of the registry with online input of data was carried out in 2020 (http://gipopt.clin-reg.ru/). The present study explored retrospective data submitted to the Russian online registry of chronic postsurgical and non-surgical hypoparathyroidism between February 2017 and June 2021. The analysis included the first data entry into the Registry (the «first visit»).

This database included all subjects affected by chronic postsurgical and non-surgical hypoparathyroidism of all ages and any etiology. The diagnosis of chronic postsurgical hypoparathyroidism was confirmed on the results of laboratory tests: persistent hypocalcemia (serum calcium levels below the lower limit of the reference range) in combination with a decreased or low-normal PTH level at least 6 months after neck surgery. The exclusion criteria include: functional hypoparathyroidism (as a result of impaired magnesium metabolism); transient postsurgical hypoparathyroidism (disease duration less than 6 months), pseudohypoparathyroidism. The term idiopathic hypoparathyroidism was used when the underlying cause was not known or had not been investigated (no available genetic tests). All clinical data were collected anonymously, using an online medical record on the registry platform. Informed consent was collected in accordance with General Authorization to Process Personal Data for Scientific Research Purposes.

The total number of patients in the registry at the time of research was 544 from 63 regions of the Russian Federation. Most of these patients were registered in Endocrinology Research centre, herewith 8 regions of the Russian Federation (the specialized endocrinological centers) have a personal access code with self-entry of data. The responsible endocrinologists from each included center submit the data online. Further it is planned to increase the number of regional connections to the registry. All clinical data in the Registry has been obtained from the baseline evaluation reported in clinical records of each center (including the Endocrinology Research Centre). The initial outpatient and inpatient medical records contain the following ICD-10 codes: E89.2 (hypoparathyroidism secondary to procedures), E20.8 (other types of hypoparathyroidism), E20.0 (idiopathic hypoparathyroidism), E20.9 (unspecified hypoparathyroidism), D82.1 (DiGeorge syndrome), E31.0 (Autoimmune polyglandular failure). Then, all data were collected and analyzed by the Endocrinology Research Centre as a coordinating institution.

The following data were analyzed: demographic variables (sex, age, geographic region); age of onset of the disease; etiology of chronic hypoparathyroidism, description of type of thyroid/parathyroid surgery (in case of postsurgical hypoparathyroidism); biochemical evaluation (serum calcium, serum phosphorus, serum magnesium, serum 25 hydroxyvitamin D (25(OH)D), serum albumin and iPTH, bone remodeling markers [alkaline phosphatase (AP), osteocalcin (OC), C-terminal telopeptide region of collagen type 1 (CTX)] and 24 h urinary calcium); genetic tests in case of non-surgical hypoparathyroidism (if available); symptoms and signs associated with hypocalcemia; long-term complications of the disease; associated disease management (used elemental calcium dose, vitamin D medication, thiazide diuretics, recombinant PTH); disease control; number of hospitalizations with acute hypocalcemia. For all laboratory parameters, the reference interval (RI) of the local laboratory was used with the obligatory indication of the lower and upper limits. The presence or absence of complications associated with chronic hypoparathyroidism was based on the results of laboratory and instrumental examination. Quantification of the bone state was carried out in the lumbar spine (L2 – L4), proximal femur [neck thigh (Neck), total hip (Total)] and radius [middle third (R33%) and radius total (RT)] using dual-energy X-ray absorptiometry (DXA) (Lunar iDXA, GE Healthcare). Bone mineral density (BMD) was assessed by Z-score. The lumbar spine trabecular bone score (TBS) was calculated on each spine DXA examination. There were no specific factors that could affect the external generalization of the research findings.

Sample size calculation principles: sample size calculation was not required.

Statistical analysis was performed using Statistica 13.0 (StatSoft, USA) and SPSS (IBM, USA) software packages. Descriptive statistics of quantitative characteristics are presented by medians and interquartile ranges (Median, IQR (25;75)%) and mean with standard deviation (SD), descriptive statistics of drug doses are presented in the form of mean, minimum and maximum values, descriptive statistics of qualitative characteristics - in absolute and relative frequencies. To analyze the correspondence of the distributions of quantitative features to the normal law, the Shapiro – Wilk test was used. The Mann – Whitney test (U-test) was applied to compare two independent groups in terms of quantitative characteristics. The critical level of significance when testing statistical hypotheses was taken equal to 0.05. In multiple comparisons, the Bonferroni correction was applied by correcting the critical level of significance. The confidence interval (CI) for frequencies was calculated by the Clopper–Pearson method. ROC-analysis was used to find cut-off points corresponding to an increase in the risk of complications. Cut-off point was selected according to the Youden’s index. Operation characteristics, such as sensitivity, specificity, positive predictive value (PPV) and negative predictive value (NPV), were calculated for the found cut-off point.

## Results

Among 544 subjects with chronic postsurgical and non-surgical hypoparathyroidism, 470 (86%) were female and 74 (14%) male. The median age of these patients was 55, IQR (41; 65) years for females and 43, IQR (24; 55) years for male. 24 subjects were under 18 years (4.4%). 6 patients were pregnant (3 patients - I trimester of pregnancy, 1 - II and 2 - III). Information about pregnancy outcomes is not available.

**Figure 1:**
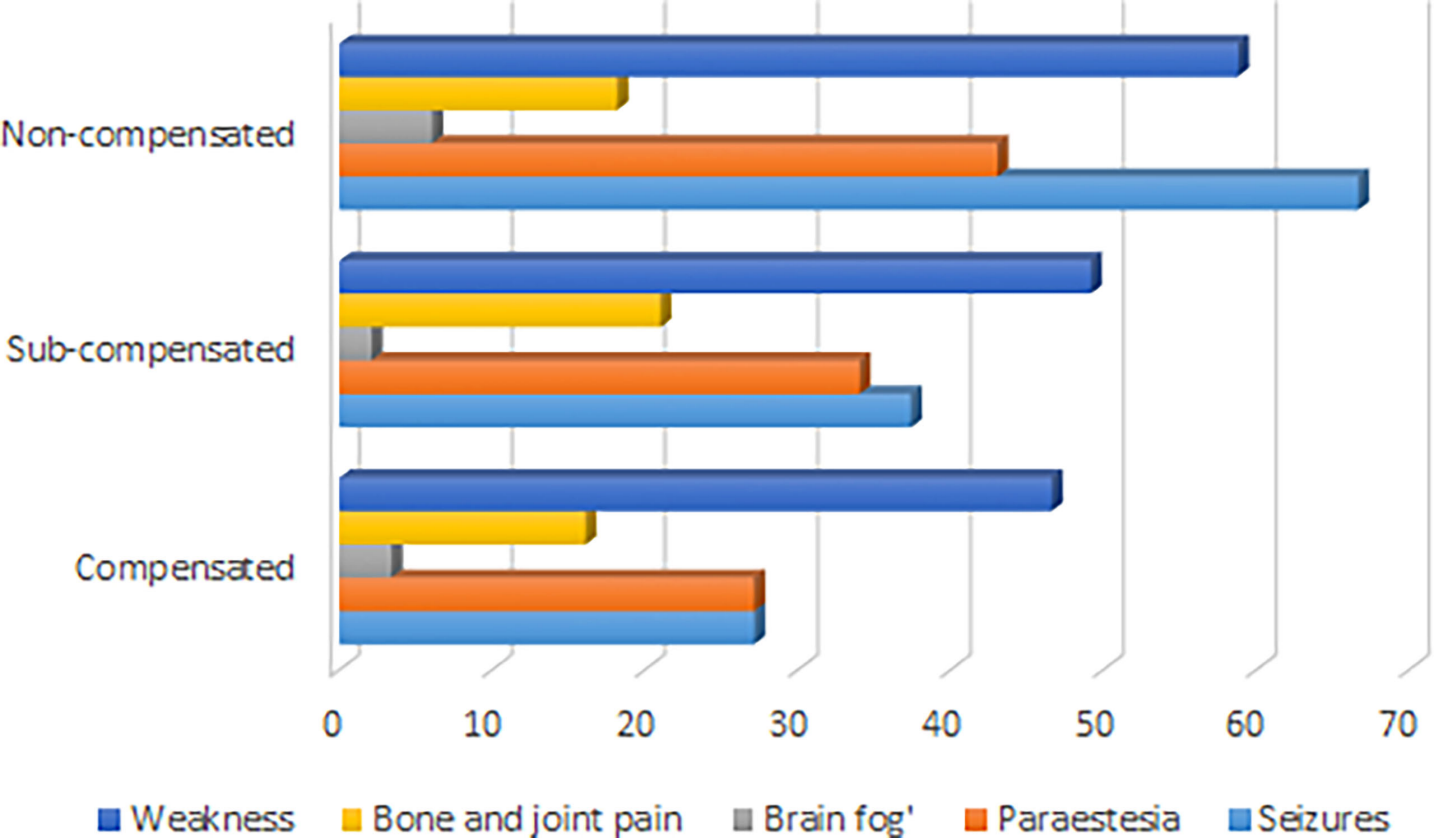
Symptoms of hypoparathyroidism according to degree of compensation of disease.

The median age of disease manifestation for patients with postsurgical hypoparathyroidism was 45, IQR (33; 55) years, while for non-surgical hypoparathyroidism, the age of manifestation was significantly lower - 19, IQR (6; 32) years (р<0.001; U-test). Postsurgical hypoparathyroidism prevailed in females (р<0.001). We did not find any gender differences for patients with non-surgical hypoparathyroidism (**Table 1**). The baseline complaints of these patients are illustrated in **Figure 1**.

**Table 1:** Demographic characteristics of group patients with hypoparathyroidism.

Characteristics		Number of patient	Number (%)		Percentage of total number, %
Sex		544	Male - 74 (13.9) Female – 470 (86.1)		100.0
Age (years)	< 18	544	24 (4.4)		100.0
	18 - 34		62 (11.4)		
	35 - 44		97 (17.8)		
	45 - 54		105 (19.3)		
	55 - 64		119 (21.9)		
	65+		137 (25.2)		
		**Etiology of hypoparathyroidism**			
		**Number of patient (%)**	**Sex, number (%)**		**Median age of disease onset, IQR (25;75) %**
			**Male**	**Female**
Postsurgical		480 (88.2)	47 (9.8)	433 (90.2)	45 (0; 83)
	Well-differentiated thyroid carcinoma	225 (46.9)	25 (11.1)	200 (88.9)
	Nontoxic unilateral/multinodular goiter	107 (22.3)	6 (5.6)	101 (94.4)
	Diffuse toxic goiter	77 (16.0)	6 (7.8)	71 (92.2)
	Hyperparathyroidism	33 (6.9)	8 (24.2)	25 (75.8)
	Others	26 (5.4)	2 (7.7)	24 (92.3)
	No data	12 (2.5)	1 (9.1)	11 (90.9)
Nonsurgical (except APS)		10 (1.8)	8 (80.0)	2 (20.0)	7 (1; 29)
Autoimmune (APS)		14 (2.6)	3 (21.4)	11 (78.6)	6 (2; 24)
Idiopathic		37 (6.8)	15 (40.5)	22 (59.5)	31 (0; 67)
Others		3 (0.6)	2 (66.7)	1 (33.3)	16 (10; 35)

APS, autoimmune polyglandular syndrome.

### Etiology

The majority of cases had postsurgical etiology (88.4%), followed by idiopathic hypoparathyroidism (6.8%), autoimmune form due to autoimmune polyglandular syndrome type 1 (1.8%), syndromic forms of genetic hypoparathyroidism (1.8%), and, finally, other forms (0.6%) (**Table 1**). Mostly the cases of postsurgical hypoparathyroidism (82.57%) occurred after a total thyroidectomy, less often after hemithyroidectomy and parathyroidectomy (in 6.6% for both). 3.1% of patients presented with chronic hypoparathyroidism after combined surgery of thyroid and parathyroid glands. All pregnant patients had postsurgical hypoparathyroidism.

The most frequent pre-surgical pathologies include thyroid cancer (49.7%), nontoxic (23.8%) or toxic goiter (17.0%). The primary and secondary hyperparathyroidism due to chronic kidney disease (CKD) accounted for 7.3% (**Table 1**).

The most causes of chronic hypoparathyroidism in age under/equal 18 years were syndromic or autoimmune forms of hereditary hypoparathyroidism (23.6%) and neck surgery (45.5%). Conversely, most causes of chronic hypoparathyroidism described over to 18 years were neck surgery (93.2%), followed by idiopathic form of hypoparathyroidism (5.9%), and, finally, non-surgical hypoparathyroidism (0.9%).

### Biochemical Data

Biochemical examination (serum calcium, phosphorus, magnesium levels and 24 h urinary calcium), collected at baseline evaluation, often showed values in non-target range, for all types of chronic hypoparathyroidism (**Table 2**).

**Table 2:** Biochemical exams of mineral metabolism for patients with hypoparathyroidism.

Target laboratory range	Number of patients	Etiology of hypoparathyroidism		Frequency, %
		Number of postsurgical patients	Number of nonsurgical patients	
Serum total calcium level, mmol/l				
All	518	455	63	95.2
< 2.1	229	208	21	44.2
2.1 - 2.3	177	156	21	34.2
> 2.3 - 2.55	94	80	14	18.1
> 2.55	18	11	7	3.5
Serum phosphate level, mmol/l				
All	420	370	50	77.2
< 0.74	1	1	0	0.2
0.74 - 1.52	219	199	20	52.2
> 1.52	200	170	30	47.6
> 2.0	31	15	16	15.5
Serum magnesium level, mmol/l				
All	233	203	30	42.8
< 0.7	55	43	12	23.6
0.7 - 1.05	177	159	18	76.0
> 1.05	1	1	0	0.4
Urinary calcium 24 h, mmol/24 h				
All	194	176	18	35.7
< 2.5	24	20	4	12.3
2.5-6.25 (for women)	74	72	2	38.1
2.5-7.5 (for men)	13	8	5	6.7
> 6.25 (for women)	73	69	4	37.6
> 7.5 (for men)	10	7	3	5.3

Among patients with available serum total calcium level only 34.2% resulted within target calcium range, and 3.5% even had true hypercalcemia.

The median serum total calcium level was 2.09, IQR (1.88; 2.26) mmol/l. The albumin-adjusted calcium level was available only for 38.8% of patients [median 2.03, IQR (1.82; 2.20) mmol/l]. The serum ionized calcium level was measured in 73.2% of patients; 45.3% showed the levels in the reference range (1.03-1.29 mmol/l), 53.3% were under 1.03 mmol/l (hypocalcemia), 1.4% were over 1.29 mmol/l. The median serum ionized calcium level accounted for 1.01, IQR (0.90; 1.10) mmol/l.

The serum phosphate level was known in 78.1% of the total cohort [1.51, IQR (1.33; 1.72) mmol/l]. Only 52.6% of patients had target phosphate levels within reference range (0.74–1.52 mmol/l). Summary hyperphosphatemia over 1.52 mmol/l was observed in 47.2%, 15.6% of patients had significant increase over 2.0 mmol/l (severe hyperphosphatemia).

The serum magnesium level was available for 45% of patients [0.74, IQR (0.7; 0.8) mmol/l]. The reference magnesium level was observed in 76% сases, under 0.7 mmol/l - in 24%, and over 1.05 mmol/l - in 0.4%.

In adult male patients (n=63) with chronic hypoparathyroidism, urinary calcium 24 h was available for 36.5% of patients; among them, 39.1% had urinary calcium levels over 7.5 mmol/24h [13.07, IQR (8.70; 14.50) mmol/24 h]. In adult female patients (n=458), this parameter was available for 41.3% and 42.3% of them had non-target indicators over 6.25 mmol/l/24h (8.95, IQR (7.59; 11.37) mmol/24h).

The level of 25(OH)D was measured in 17.43% of the patients with chronic hypoparathyroidism and about half of them were insufficient [less than 30 ng/mL in 46.31%].

### Complications

#### Severe Acute Hypocalcemia

In this study 11 patients (8 with postsurgical hypoparathyroidism, 1 - autoimmune form due to autoimmune polyglandular syndrome type 1 and 2 – idiopathic hypoparathyroidism) had hospitalization due to severe acute hypocalcemia during the last year. Most of these patients had 1-2 acute hospitalizations during the year, but 2 patients (both patients with postsurgical hypoparathyroidism) had 6 and 10 hospitalizations with severe acute hypocalcemia during the year.

#### Kidney

Ultrasound and computed tomography (CT) kidneys were performed in 39% of patients. In the examined group, nephrolithiasis was confirmed in 32.5%, nephrocalcinosis - in 12.3%. Nephrocalcinosis was more often bilateral (74% of cases). 15.8% of patients with chronic hypoparathyroidism met the criteria of CKD 3a-5 stage (glomerular filtration rate (GFR)-EPI less than 60 ml/min/1.73m2).

The serum total and ionized calcium levels have negatively correlated with GFR (p=0.0008 and p=0.0002 respectively, Bonferroni correction Р0 = 0.0071). We did not reveal a significant association between GFR and serum phosphorus level (p=0.0197). GFR was lower in patients with a longer duration of the disease (p=0.0057, Bonferroni correction Р0 = 0.0071), but it did not correlate with the presence of nephrolithiasis/nephrocalcinosis and with the medical therapy generally as well as with dosing regimen (calcitriol, alfacalcidol, calcium supplements and hydrochlorothiazide were taken into account).

**Figure 2:**
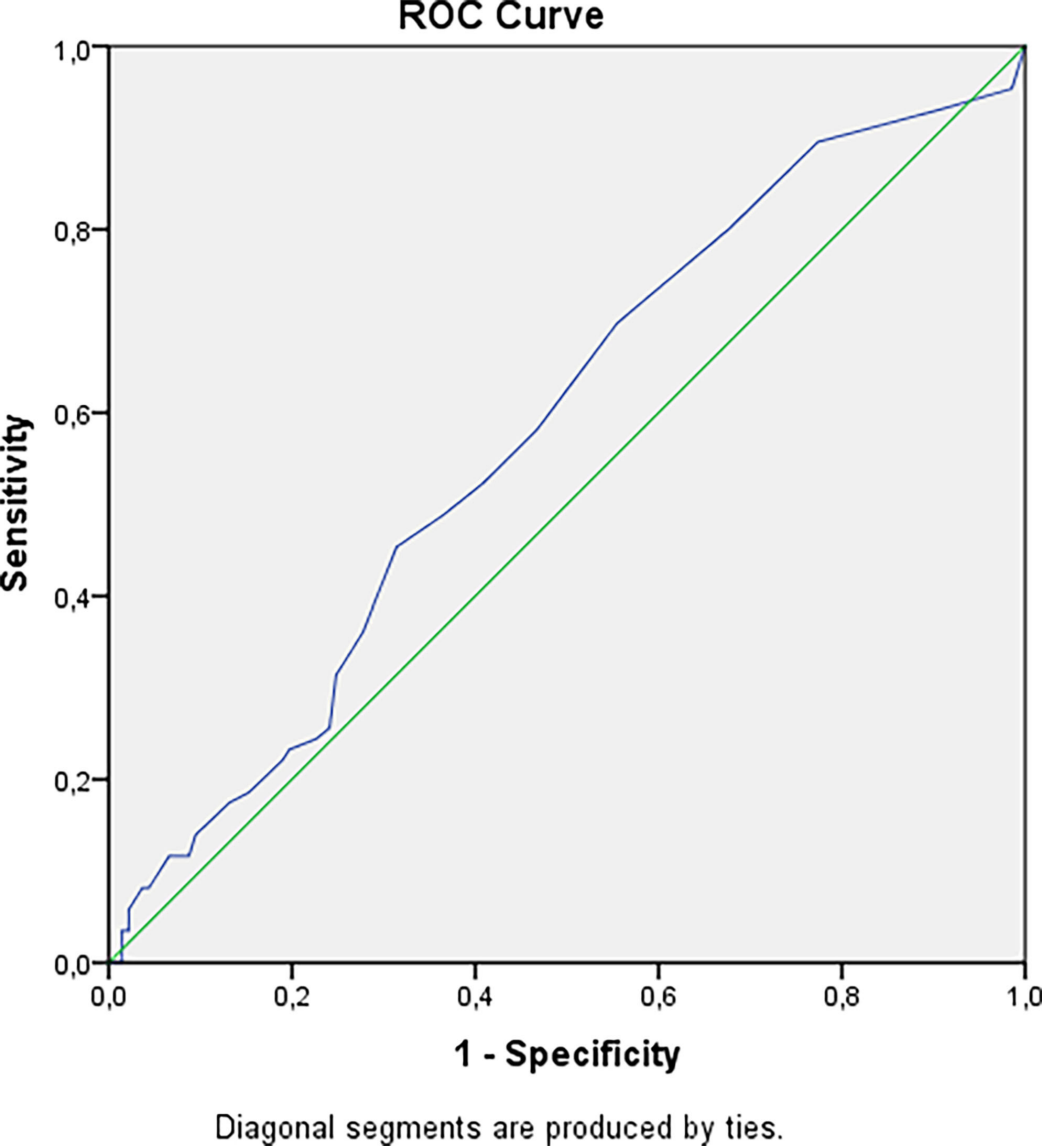
Cut-off point for developing nephrolithiasis/nephrocalcinosis in patients with hypoparathyroidism. AUC=0.582 (95% CI: 0.505; 0.658), р=0.040. Cut-off point – 4.5 years. Sensitivity = 70% (95% CI: 61%; 78%). Specificity = 45% (95% CI: 39%; 50%). PPV = 44% (95% CI: 39%; 49%). NPV = 70% (95% CI: 62%; 78%). OR = 1.85 (95% CI: 1.05; 3.28).

**Figure 3:**
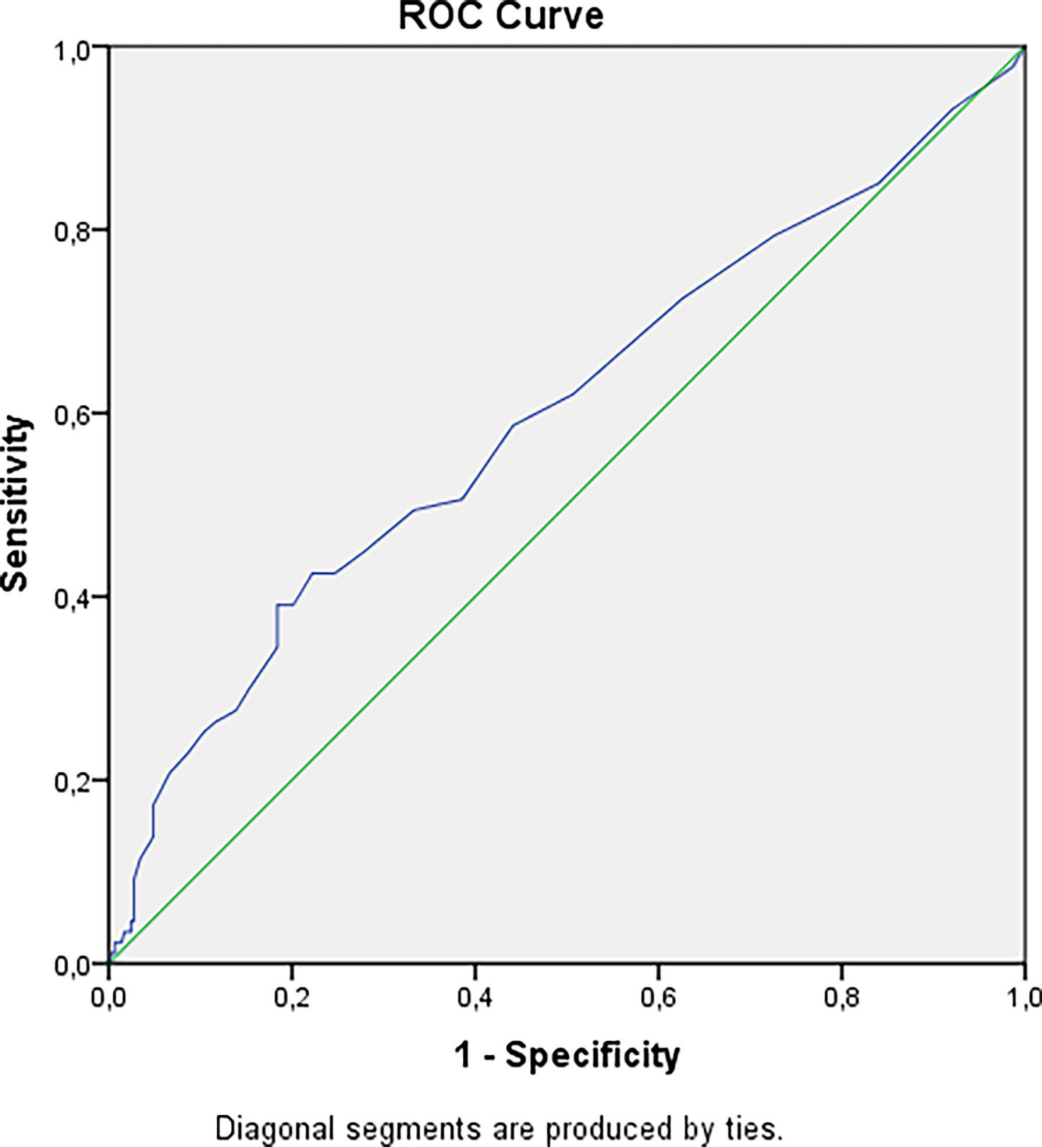
Cut-off point for decreasing GFR in patients with hypoparathyroidism. AUC=0.598 (95% CI: 0.526; 0.670), р=0.006. Cut-off point – 13.5 years. Sensitivity = 39% (95% CI: 30%; 48%). Specificity = 82% (95% CI: 79%; 84%). PPV = 39% (95% CI: 30%; 48%). NPV = 82% (95% CI: 79%; 84%). OR = 2.84 (95% CI: 1.69; 4.80).

We have revealed a statistical tendency that in patients with a higher urinary calcium 24h level and longer duration of disease nephrolithiasis is more often detected (p=0.0167 and p=0.0405 respectively, Bonferroni correction Р0 = 0.0071). The risk of nephrocalcinosis/nephrolithiasis increased by 1.85 times with disease duration more than 4.5 years (**Figure 2**). The cut-off point for the decreasing GFR was 13.5 years, with a 2.84-fold increased risk (**Figure 3**).

#### Brain

Native CT scan of the brain was available in 44 patients (8%), among them basal ganglia calcification was detected in more than half of these cases (54.5%). The patients with Fahr’s syndrome suffered from neuropsychiatric disease in 34.5% cases.

We have found that patients with longer duration of hypoparathyroidism have more often calcification in the brain (p=0.0018, Bonferroni correction Р_0 =_ 0.0071). There was no association with standard therapy with calcium or vitamin D supplements, as well as with thiazide diuretics. Patients with non-surgical hypoparathyroidism have a tendency to higher frequency of brain calcification compared to postsurgical hypoparathyroidism (р=0.0331).

#### Neuropsychiatric Disturbances

The neuropsychiatric disturbances have been diagnosed in 28 patients (5.15%, 20 patients with postsurgical hypoparathyroidism, 2 – autosome-dominant hypocalcemia, 1 patient with DiGeorge syndrome and 5 with idiopathic form of hypoparathyroidism) and they followed up by psychiatrists, among them 6 patients suffer from with depression, 4 - from dysthymia, 4 - from with dyscirculatory encephalopathy and 14 from other neuropsychiatric diseases.

#### Ophthalmological Disease

**Figure 4:**
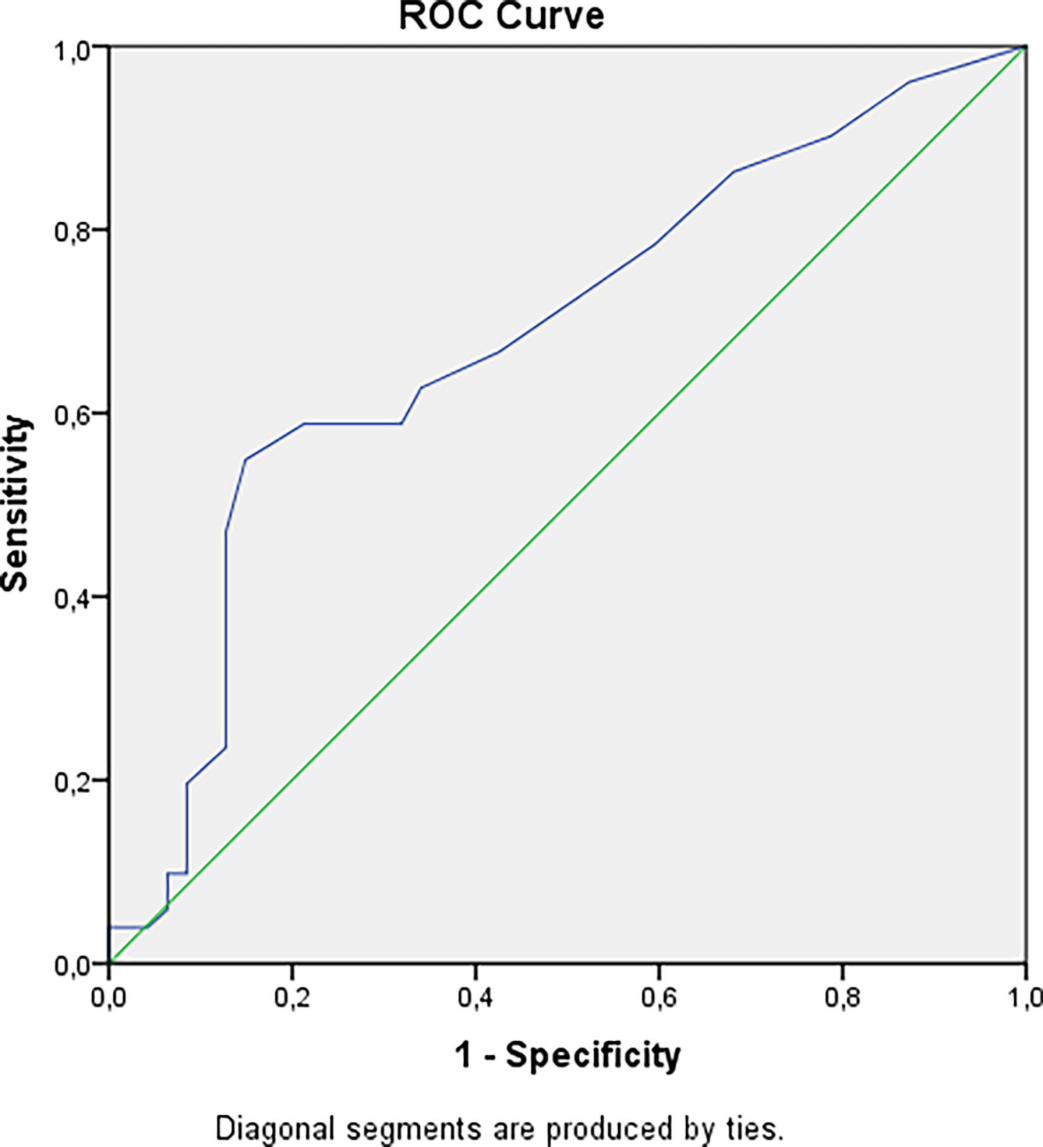
Cut-off point for developing cataract in patients with hypoparathyroidism. AUC=0.684 (95% CI: 0.576; 0.791), р=0.002. Cut-off point – 9.5 years. Sensitivity = 55% (95% CI: 45%; 62%). Specificity = 85% (95% CI: 75%; 93%). PPV = 80% (95% CI: 66%; 90%). NPV = 64% (95% CI: 56%; 69%). OR = 6.96 (95% CI: 2.63; 18.43).

The cataract was confirmed in 51 (9.4%) patients with hypoparathyroidism, 80.4% of these patients had postsurgical hypoparathyroidism. The longer duration of hypoparathyroidism was associated with more frequent cataract (p=0.0018, Bonferroni correction Р_0 =_ 0.0071) and this does not depend on drugs and their doses. The cut-off point for the development of cataracts was 9.5 years, with a 6.96 -fold increased risk (**Figure 4**), after adjustment for age OR= 5.40 (95% CI: 1.82; 16.0).

#### Heart Disturbances

Electrocardiography was performed in 168 patients. The rhythm disturbances were confirmed in 39 patients and dilated cardiomyopathy - in 1 patient. The average duration of hypoparathyroidism in patients with arrhythmia was variable (mean duration: 9 years, min: 1 year and max: 43 years). All patients with cardiac rhythm disturbances were non-compensated.

#### Bone Status

The DXA with TBS assessment was performed only in 66 adult patients (12.1%). Generally the BMD in 3 areas (spine, hip and radius) corresponded to age values, and there was no evidence for the phenomenon of high bone density. TBS was consistent with normal bone microarchitectonics (**Table 3**). 26 (4.8%) patients underwent low-trauma fractures in different parts of the skeleton. 8 patients had vertebral fractures, 5 - ankle fractures, 10 - in bones of the upper limb and 3 in pelvic. The level of AP was available for 29.8% (RI 40-150 ui/l) of the total database, OC (RI 15-46 ng/ml; N: 78) and CTX (RI 0.3-1.1 ng/ml; N:71) for 14.3 and 13.1% respectively. In adult patients with chronic hypoparathyroidism the mean value of bone turnover markers was low to normal: 66, IQR (50; 87) iu/l for AP; 14.35, IQR (9.86; 18.51) ng/ml for OC and 0.22, IQR (0.14; 0.36) ng/ml for CTX (**Table 3**). Interestingly, the serum AP levels were higher in younger patients (less than 18 years) compared to adult patients with non-surgical hypoparathyroidism (p=0.0001, Bonferroni correction Р0 = 0.0250). Moreover there was a tendency between the longer duration of the disease and lower serum AP level (p=0.0140, Bonferroni correction Р0 = 0.0014). We have not found any correlation between duration disease and other bone mineral markers (OC, CTX) as well as bone mineral markers (AP, OC, CTX) and BMD, Z-score and TBS.

**Table 3:** Bone turnover markers, bone mineral density (BMD) and Z-score in patients with postsurgical and non-surgical hypoparathyroidism.

Bone turnover markers	Median, IQR (25;75)% for patients with postsurgical hypoparathyroidism		Median, IQR (25;75)% for patients with non-surgical hypoparathyroidism		p, U-test
**Bone turnover markers**				
AP (units/l)	62 [49; 79]		83.9 [66; 161.5]		<0.001
OC (ng/ml)	13.65 [9.76; 17.52]		15.89 [14.78; 23.11]		0.027
CTX (ng/ml)	0.2 [0.135; 0.305]		0.36 [0.24; 0.38]		0.044
**BMD, TBS and Z-score**				
**Parameters**	**Median, IQR (25;75)% for patients with postsurgical hypoparathyroidism**		**Median, IQR (25;75)% for patients with non-surgical hypoparathyroidism**		
Years	18-50 years	>50 years	>=18 years		
L2-L4 BMD	1.333 (1.221; 1.445)	1.224 (0.947; 1.341)	1.173 (1.043; 1.304)		
L2-L4 SD (Z-score)	1.1 (-0.5; 2.3)	0.4 (-2.1; 2.2)	1.4 (0.6; 3.3)		
Femur Neck BMD	1.034 (0.968; 1.077)	0.955 (0.844; 1.108)	1.078 (1.042; 1.114)		
Femur Neck SD (Z -score)	0 (-0.6; 1.0)	0 (-1.6; 0.8)	0.75 (0.6; 1.6)		
Total hip BMD	1.084 (1.006; 1.192)	1.087 (0.941; 1.231)	1.07 (0.996; 1.144)		
Total hip SD (Z-score)	0.4 (-0.3; 1.8)	1.4 (0.7; 2.0)	0.6 (0.3; 1.9)		
Radius Total BMD	0.703 (0.677; 0.751)	0.708 (0.600; 0.774)	0.73 (0.643; 0.817)		
Radius Total SD (Z-score)	0.2 (-0.3; 1.0)	1.3 (0; 2.6)	0.95 (0.2; 1.0)		
Radius 33% BMD	0.888 (0843; 0.929)	0.910 (0.734; 0.977)	0.891 (0.868; 0.915)		
Radius 33% SD (Z-score)	0.1 (-0.4; 0.4)	-0.3 (-3.4; 1.0)	-0.2 (-0.5; 0.45)		
TBS	1.53 (1.39; 1.58)	1.42 (1.34; 1.54)	1.48 (1.37; 1.64)		

The duration of the disease showed positive correlations with Z-score in the axial skeleton - L2-L4, femur neck and total hip (p=0.0006, p=0.0008 and p=0.0084 respectively, Bonferroni correction Р0 = 0.0009), but not in radius total and radius 33%.

### Medical Therapy

The medical therapy was known in 461 patients (85%), the majority (83.5%) was treated with calcium and vitamin D supplements (metabolites and analogs). The average dose of alfacalcidol was 1.7 mcg/day (min: 0.25, max: 8.0); calcitriol - 1.3 mcg/day (min: 0.25, max: 5.0); elemental calcium – 1800 mg/day (min: 250, max: 8000). 102 patients (22%) required more than 2,500 mg/day of elemental calcium per day and 28 (6%) - more than 3 mcg of alfacalcidol. The cholecalciferol was taken by 48% (n = 222) of patients with mean doses 2800 IU/day (min: 100, max: 5000).

Among pregnant patients 5 were treated by a combination with alfacalcidol [the average dose was 1.9 mcg/day (min: 0.5, max: 4.0)] and calcium carbonate [the average dose was 1220 mg/day (min: 500, max: 2500)] and 1 women - cholecalciferol (2000 IU/day) and calcium carbonate (1000 mg/day).

Additional treatments (magnesium and potassium medication, thiazide diuretics and recombinant PTH) were prescribed to 13% of patients. Magnesium supplementation was taken by 11.3% (n = 52); thiazide diuretics - by 10% individuals with hypercalciuria [a mean dose 25 mg/day (min: 6.25, max: 75)].

29% of patients (n=134) have achieved total biochemical control - target serum calcium and phosphate levels, normocalciuria. 41% of patients (n=193) were sub-compensated with a slow decrease in serum calcium levels. 35.8% (n=165) had an uncontrolled disease with severe hypocalcemia. 14 patients were taking an inhibitor proton pump and most of them were decompensated (95%).

21 patients (3.9%) were treated with dihydrotachysterol at the time of inclusion to the Registry. Most of these patients were non-compensated (95%). In all cases the therapy with dihydrotachysterol was changed to alfacalcidol.

5 subjects (women/men – 4/1) with severe hypoparathyroidism and the need for high doses of standard therapy have received teriparatide [rhPTH (1–34)] with mean dose 28 mcg/day (min: 20, max: 40) in addition to alfacalcidol [mean dose 2 mcg/day (min: 1.0, max: 3.0)/calcitriol (4 mcg/day)] and calcium supplementation (3000-4000 mg/day). In one case, teriparatide was used in pump infusion. 4 patients had postsurgical hypoparathyroidism and one patient - idiopathic hypoparathyroidism.

## Discussion

The revealed predominance of postsurgical hypoparathyroidism, especially among young women at reproductive age, are consistent with the studies from the USA, European and other countries as well as with the first study on Russian population (8–10). This is most likely associated with both prevalence of thyroid pathology in this population and a greater adherence to examination and, consequently, a higher frequency of neck surgery. The volume of surgical intervention is an independent risk factor for the development of chronic hypoparathyroidism; therefore, thyroid cancer and nontoxic goiter predominate in the structure of preoperative pathology (11).

Currently special attention is paid to the problem of “uncontrolled” hypoparathyroidism, not only associated with deviations in the calcium-phosphorus levels, but also with long-term complications and their predictors, substantial symptoms and decreased patient health-related QoL (12). In this study, about half of the patients had targets for serum calcium (34% - 2.1-2.3 mmol/l, 22% - >2.3 mmol/l) and phosphorus (52% in RR). The obtained data confirm the absence of adequate disease control in a large percentage of patients and the need for timely monitoring and medical treatment (8, 12, 13). The existing recommendations for the management of patients with chronic hypoparathyroidism do not have clear recommendations on the timing of examination for the disease complications of the disease. The analysis of large databases especial with the long-term follow-up can help to create the evidence-based recommendations on this subject.

The renal impairment is the most common long-term complication of chronic hypoparathyroidism. Аccording to different cohort studies, the frequency of nephrocalcinosis/nephrolithiasis varies from 12 to 57%, while the risks of their manifestation are 5 times higher than in the general population (14, 15). Similar results were noted for renal function. The retrospective data from the Massachusetts General Hospital demonstrated that the frequency of CKD stage 3-5 in patients with hypoparathyroidism was 2-fold to 35-fold greater than age-appropriate normal values. Renal failure with a decrease in GFR less than 60 ml/min/1.73 m^2^ was recorded in 12–41% of cases. The other studies from Denmark showed the threefold higher risks of the renal insufficiency in postsurgical hypoparathyroidism [hazard ratio (HR) 3.10, 95% CI 1.73–5.55] and six fold higher risks in non-surgical hypoparathyroidism (HR 6.01, 95% CI 2.45–14.75). Renal stones were found more frequent in postsurgical hypoparathyroidism (HR 4.02, 95% CI 1.64–9.90) after adjustment for prior diabetes and renal disease (16). In our study 39% of patients had renal disease imaging, among them nephrolithiasis or nephrocalcinosis were detected in 32.6 and in 12.3% respectively, an GFR less than 60 mL/min/1.73 m2 (CKD grade 3-5) was observed in 15.8% of patients. According to our results, the longer duration of disease is crucial for the risks of nephrocalcinosis/nephrolithiasis and CKD with decreased GFR regardless of the hypoparathyroidism etiology. The cut-off points were 4.5 and 13.5 respectively.

Hypercalciuria is generally considered to be the most common metabolic risk factor for calcium nephrolithiasis. Calcium oxalates and/or phosphates are predominant renal stones comprising about 80-90% of all urinary calculi (17). Patients with hypoparathyroidism due to lack of PTH effects, may have an increased level of urinary calcium. In this instance hypercalciuria is associated with elevated blood calcium levels (the concentration of calcium in urine increases by an average of 50 mg/day for each increase in serum calcium level by 1 mg/dL, P<0.001) (14). We did not confirm a significant association between nephrocalcinosis/nephrolithiasis and hypercalciuria, only the tendency, but one of the reasons may be insufficient examination of patients.

The association between hypoparathyroidism and cataract, primarily of the posterior subcapsular type, was described for non-surgical hypoparathyroidism. Underbjerg et al. showed the increased risks to develop cataracts in non-surgical hypoparathyroidism versus control group (HR 4.21, 95% CI 2.13–8.34), with a younger age of onset (53 vs 60 years). There was no difference between patients with postsurgical hypoparathyroidism and controls in incidence (HR 1.17, 95% CI 0.66–2.09) or age of onset (5, 18). In the present study, cataract was diagnosed in 9.4% of patients, there was no difference in postsurgical and non-surgical groups (р=0.483).This is likely to be explained due to the high disease duration in both groups. The cut-off point for the development of cataracts was 9.5 years, with a 6.96-fold increased risk, after adjustment for age OR= 5.40.

Basal ganglia calcification is a severe complication of chronic hypoparathyroidism and can be observed in 52–74% of cases (19–21). In the present study, 8% of patients underwent the CT Scan of the Head/Brain at baseline evaluation, where basal ganglia calcification was revealed in more than in a half of cases. The risks of developing neuropsychiatric disorders are 2.01–2.45 times higher than in the general population (18, 22, 23). In our cohort, the common prevalence of neuropsychiatric disorders was 5.15%, although this is most likely due to the low referral to a psychiatrist.

Both hypo- and hypercalcemia increase the risk of cardiac arrhythmias. In case of hypoparathyroidism the rhythm disorders develop more often as a result of acute hypocalcemia, which causes QT prolongation. However, these arrhythmias are generally reversible after reaching target calcium values. According to our results, the incidence of this complication was 7.17%. Additionally, chronic hypocalcemia can cause dilated cardiomyopathy, but it is an uncommon condition (24).

Bone disease occurs in hypoparathyroidism due to the absence or low levels of PTH and so markedly reduced bone remodeling. Low levels of PTH lead to a low bone turnover state, usually resulting in increased BMD on DXA than in age- and sex-matched controls (14, 25) as well as in microarchitectonics changes confirmed by bone biopsies, micro-CT, peripheral quantitative CT and high-resolution peripheral quantitative CT (26). Results from the study by Rubin et al. confirmed the increase in cancellous bone volume and trabecular thickness in hypoparathyroid subjects, and demonstrated higher trabecular number and trabecular connectivity in comparison with matched control subjects. In addition, the structural model index is lower in hypoparathyroidism (27). Whether this increased BMD influences on fracture risk is less certain, because while increased bone mineralization may be associated with increased brittleness of bone, this does not appear to be the case in hypoparathyroidism (25, 28).

Unexpected, but in our work, the BMD values also remained within the normal range in all parts of the skeleton which is likely to be due to the initial heterogeneity of the studied group at the time of the first entry of data into the register map - the different duration of disease, gender, age and others. We have not analyzed other facts that can affect the BMD such glucocorticoid intake, diabetes mellitus, smoking, which is a limitation of the current study. At the same time, Sakane et al. have observed that the most patients with postsurgical hypoparathyroidism had had the DXA findings in the normal range but the time elapsed from surgery positively correlated with BMD values. We have also showed that the duration of the disease had a positive correlation with Z-score in the axial skeleton - L2-L4, femur neck and total hip but not in the Radius.

TBS has been previously validated as a tool to estimate bone quality in several conditions especially in postmenopausal osteoporosis. Cipriani et al. evaluated the TBS of patients with various causes of chronic hypoparathyroidism. The mean TBS values in their population remained in normal range, 1.44 ± 0.12 (29), consistent with our results, even when women were divided into postmenopausal and premenopausal groups. In the study of Sakane et al. the mean TBS value was 1.386 ± 0.140, 32.2% of the results were under than 1.310. TBS values correlated negatively with BMI, age, and glycaemia, whereas abnormal TBS correlated with osteopenia, diabetes mellitus 2, low-impact fracture and menopause. Thus, the authors concluded that factors related to loss of bone mass in the general population also can affect the bone microarchitecture in hypoparathyroidism (30).

In our work, the BMD values ​​also remained within the normal range in all parts of the skeleton; TBS demonstrated preserved microarchitectonics. However, the mean value of bone turnover markers was low to normal which is consistent with the results of other studies (31).

Conventional therapy is first-line therapy and based on calcium and vitamin D supplementation. Dietary calcium is frequently suboptimal in the healthy adult population, and even more likely to be inadequate for managing chronic hypoparathyroidism without calcium supplementation. Up to 83-93% of patients with chronic hypoparathyroidism adhere to this therapy, usually, the daily dosage of alfacalcidol/calcitriol ranges from 0.5 to 3.0 mcg, calcium supplements - 1000–2000 mg/day (32). In our study, the majority of patients (83.5%) was treated with calcium and vitamin D supplements (metabolites and analogs). 22% required more than 2,500 mg/day of elemental calcium per day and 6% - >3 mcg of alfacalcidol or calcitriol. Patients are also typically supplemented with either of the two parental forms of vitamin D (cholecalciferol). The rationale for using these forms of vitamin D is that many tissues generate their own 1,25-dihydroxyvitamin D and other metabolites of vitamin D that may have beneficial nonskeletal effects (33, 34). In our study, the cholecalciferol was taken by 48% of patients with mean doses 2800 IU/day (min: 100, max: 5000).

Dihydrotachysterol should not be used for the treatment of chronic hypoparathyroidism first of all due to the high risks of hypercalcemic episodes (up to hypercalcemic crisis). The other side effects of the drug include nephrocalcinosis and progressive renal failure (35, 36). In some cases, patients were treated non-rationale – 3.13% only salt of calcium and 1.1% only native form vitamin D (cholecalciferol).

The replacement therapy in chronic hypoparathyroidism could be an option for patients with a severe course of disease. The FDA approved once-daily rhPTH (1–84) for adjunctive therapy of chronic hypoparathyroidism in adults, later this drug received approval by the EMA. Unfortunately, rhPTH (1–84) was recalled by the FDA in 2019, due to technical problems with the delivery device and the recall is expected to remain in force through mid-2022. Only Teriparatide (PTH (1–34)) is the recombinant PTH available in the Russian Federation. It is registered for the treatment of osteoporosis, but there is long-term data from research studies regarding PTH (1–34) treatment in adults and children with hypoparathyroidism (37). There is a positive 10 years experience of using teriparatide with multiple daily injections in 14 children with chronic non-surgical hypoparathyroidism. There was a significant decrease in the need for calcium and vitamin D supplements, as well as a positive dynamics in bone remodeling markers. However, further randomized clinical trials are required to assess the safety of therapy in different age groups of patients. In our study, 5 patients were treated with rhPTH (1–34) by decision of the medical council in the specialized endocrinological centers. Teriparatide was prescribed only in case of poor disease control and life-threatening hypocalcaemia despite large doses of calcium and vitamin D supplements. Difficulties in achieving target calcium levels were associated more often with malabsorption syndrome. The decision was based on the order of the Ministry of Health and Social Development of the Russian Federation of 08.09.2005 N494 (“The use of drugs in patients according to vital indications”).

### Limitations

There is a retrospective study with the limitations inherent to the loss of data inserted to the Registry platform. Moreover, there is a multicenter study. The labor parameters were determined in different laboratories, as well as instrumental studies were performed by various specialists on different equipment. The Russian clinical guidelines for hypoparathyroidism were created and approved only in 2021, thus there was no official standard for examining patients. Moreover due to the widely lack of recommendations for complications screening (frequency of ophthalmic examination, СT of brain, regular electrocardiography), the most patients had no data about these complications. The extended examination in presented study was performed only in hospital patients mostly in Endocrinology Research Centre.

## Conclusion

This study represents the one of the first large-scale epidemiological assessments of chronic hypoparathyroidism in the Russian Federation. Analysis of the presented database indicates insufficient diagnosis of the complications in patients with chronic hypoparathyroidism, especially with regard to long-term complications. Overall, hypoparathyroidism and its long-term standard therapy are associated with higher risks of renal stone formation, decreased GFR, especially in those with episodes of treatment-induced hypercalcemia, ectopic calcifications, impaired QoL. Noteworthy is a large percentage of patients with inadequate treatment options, which indicates insufficient therapy and lack of control over the disease. The analysis of large databases of patients with chronic hypoparathyroidism is a necessary tool to enhance quality of medical care, as well as to determine the optimal clinical and therapeutic approaches, prognostic markers of disease complications.

## Data Availability Statement

The datasets presented in this article are not readily available because this database is copyrighted. Requests to access the datasets should be directed to Elena V. Kovaleva, elen.v.kovaleva@gmail.com.

## Ethics Statement

The studies involving human participants were reviewed and approved by the Ethics Committee (Protocol No. 18 dated October 11, 2017). Written informed consent to participate in this study was provided by the participants or participants’ legal guardian/next of kin.

## Author Contributions

GM and NM contributed to conception and design of the study. EK, AKE, ARE, JK, EB, IM, AG, and ED organized the database. ARE performed the statistical analysis. EK and AKE wrote the first draft of the manuscript. JK, EB, IM, AG, and ED wrote sections of the manuscript. All authors contributed to manuscript revision, read, and approved the submitted version.

## Funding

The study was supported by the Ministry of Science and Higher Education of the Russian Federation (agreement no. 075-15-2020-899).

## Conflict of Interest

The authors declare that the research was conducted in the absence of any commercial or financial relationships that could be construed as a potential conflict of interest.

## Publisher’s Note

All claims expressed in this article are solely those of the authors and do not necessarily represent those of their affiliated organizations, or those of the publisher, the editors and the reviewers. Any product that may be evaluated in this article, or claim that may be made by its manufacturer, is not guaranteed or endorsed by the publisher.
